# Predictive Value of Acute Phase Proteins for the Short-Term Outcome of Meningoencephalitis of Unknown Origin in Dogs

**DOI:** 10.3390/ani13162575

**Published:** 2023-08-10

**Authors:** Aurora Cocchetto, Andrea Zoia, Rita Aragão, Laura Ventura, Marika Menchetti

**Affiliations:** 1 Division of Neurology and Neurosurgery, San Marco Veterinary Clinic, Viale dell’Industria 3, 35030 Veggiano, Italy; 2 Division of Internal Medicine, San Marco Veterinary Clinic, Viale dell’Industria 3, 35030 Veggiano, Italy; 3 Department of Statistical Sciences, University of Padova, Via Cesare Battisti 241, 35121 Padova, Italy

**Keywords:** biomarkers, *Canis lupus familiaris*, dogs, inflammation, inflammatory brain disease, central nervous system

## Abstract

**Simple Summary:**

Meningoencephalitis of unknown origin is a heterogeneous group of inflammatory diseases of the central nervous system, accounting for the vast majority of the immune-mediated condition of the central nervous system. The prognoses of these diseases are variable, and it is crucial to discover reliable and easy-to-detect biomarkers for predicting the outcome. This will help clinicians and pet owners make informed decisions about their pets’ care. This retrospective study aims to evaluate the potential role of various routinely assessed serum inflammatory parameters as biomarkers predicting the short-term outcome in patients newly diagnosed with meningoencephalitis of unknown origin. Based on the short-term outcome, the patients were classified into one of two groups: survived and non-survived dogs. None of the parameters evaluated was able to predict the outcome. Additionally, none of these variables showed a consistent increase in the current group of dogs, regardless of the result. In conclusion, based on the results of the present study, meningoencephalitis of unknown origin is not associated with a detectable systemic inflammatory condition, and routinely assessed serum inflammatory parameters are not a useful tool to predict the short-term outcome of this disease.

**Abstract:**

Meningoencephalitis of unknown origin (MUO) is one of the most common inflammatory diseases of the central nervous system (CNS). The study evaluates the possible increase and the potential role of acute phase proteins (APPs) and other inflammatory serum parameters as biomarkers predicting the short-term outcome of dogs with meningoencephalitis of unknown origin (MUO). A retrospective cohort study was designed. The APP profile and other markers of systemic inflammation of forty-eight client-owned dogs with a new diagnosis of MUO were compared between 7-day survival and non-survival dogs diagnosed with MUO. Thirty-nine (81%) dogs were alive at the end of the 7-day follow-up period, while 9 (19%) dogs died or were euthanized because of MUO. None of the 11 markers of inflammation studied were different between the survived and non-survived dogs; for this reason, none of them could be used as a predictor of the short-term outcome based on the results of the present study. This confirms that even though MUO is often associated with a severe inflammatory status of the central nervous system (CNS), this condition is probably isolated exclusively to the CNS.

## 1. Introduction

Meningoencephalitis of unknown origin (MUO) is one of the most common inflammatory diseases of the central nervous system (CNS), accounting for 47.5% of immune-mediated conditions of the CNS in dogs [1]. MUO is a group of diseases subclassified into granulomatous meningoencephalitis (GME), necrotizing meningoencephalitis (NME), and necrotizing leucoencephalitis (LME). Young to middle-aged toy and small-breed dogs are predisposed to MUO [2]; however, dogs of any age and breed can be affected [3], and 25% of the dogs most commonly affected are large-breed, according to a recent study [4]. Neurological clinical signs could be focal or multifocal and depend on the location of the lesions within the CNS. Systemic signs, such as fever and leukocytosis, are uncommon [3].

The pathogenesis of MUO is considered multifactorial and the main hypothesis for the etiology is an autoimmune cause triggered by environmental or infectious events [5,6,7,8,9,10]. The diagnosis of MUO is presumptive and magnetic resonance imaging (MRI), cerebrospinal fluid (CSF) analysis, and the exclusion of infectious agents are used to support the suspicion, although the definitive diagnosis is made only by histopathology [2,3,11]. Treatment involves using steroids with other immunosuppressive agents [12,13,14,15,16,17,18].

The prognosis of MUO is variable [19]. In the majority of cases, death occurs within the first week after the diagnosis [13,20] and dogs surviving more than three months after diagnosis have a low risk of death because of MUO [13].

Biomarkers are mostly endogenous or introduced substances that can be measured in the body and can predict the incidence or outcome of a disease [21]. They are useful tools, especially when they are low-cost and easy to detect (for example, in serum or plasma), so they can be introduced in the daily diagnostic investigation. Several biomarkers have been proposed during the last several decades. The CSF’s concentrations of neutrophil gelatinase-associated lipocalin (NGAL) and microRNA are higher in dogs with MUO than in dogs with other non-inflammatory CNS diseases [22,23]. Higher blood and CSF lactate concentrations have proved to be associated with shorter survival and more severe neurologic signs in dogs with MUO in one study [24]. Furthermore, serum and CSF neurofilament light chain protein concentrations are higher in patients with MUO and in dogs with a poor outcome despite the treatment compared to healthy dogs and dogs responding well to the immunosuppressive treatment for MUO [25].

Acute phase proteins (APPs) are serum proteins that change in concentration in response to systemic inflammation [26], and they are highly sensitive indicators of inflammation but with low specificity regarding the underlying cause [26]. Until now, there has been limited evidence of their behavior in MUO [21]; hence, this retrospective cohort study aims to investigate the behavior and the potential role of APPs and other inflammatory serum parameters as biomarkers predicting the short-term outcome in dogs diagnosed with MUO.

## 2. Materials and Methods

The study population included forty-eight client-owned dogs. Previous informed written consent was obtained. All the procedures performed were made solely for the dogs’ benefit and standard diagnostic and monitoring purposes; they also complied with the European legislation “on the protection of animals used for scientific purposes” (Directive 2010/63/EU) and with the ethical requirement of the Italian law (Decreto Legislativo 4 March 2014, n. 26). Accordingly, this study did not require authorization or an ID protocol number.

### 2.1. Case Selection

The electronical medical database of San Marco Veterinary Clinic was searched and reviewed for cases of newly diagnosed MUO from May 2017 to December 2021.

### 2.2. Inclusion Criteria

The diagnostic inclusion criteria for MUO were based on the study by Granger et al. [2] with minor modifications. Cases with a presumptive diagnosis of MUO were included if all of the following criteria were observed: (a) dogs were >6 months of age, (b) focal or multifocal CNS neurologic signs were observed, (c) an MRI study was performed, and (d) hyperintense lesions were observed on T2-weighted (T2W) and fluid-attenuated inversion recovery images (FLAIR), or (e) MRI lesions were not observed but (f) CSF abnormalities (total nucleated cell count (TNCC) > 5 cell/μL and pleocytosis with >50% mononuclear cells (monocytes and lymphocytes)) were noted and (g) the presence of infectious diseases was ruled out. Moreover, dogs with a presumptive diagnosis of MUO and with MRI lesions were included only if CSF abnormalities were noted or (h) a CSF tap was not performed or was normal but the MRI lesions matched those most commonly seen in MUO cases, according to the study by Granger et al. [2] (i.e., multiple, single, or diffuse intra-axial hyperintense lesions on T2W MRI) and (i) the presence of infectious diseases was ruled out. The infectious diseases that were tested for, either by CSF or blood PCR, included *Toxoplasma gondii*, *Leishmania infantum*, *Neospora caninum*, and canine distemper virus. Furthermore, the cases to be included needed a complete medical record available for review, including history, general physical examination, neurological examination, complete blood count (CBC), full biochemistry panel, urinalysis, and coagulation profile, all collected at the time of diagnosis. All the included dogs received immunosuppressive steroid therapy alone or with one or more immunosuppressants, starting at the time of the diagnosis of MUO or shortly thereafter.

### 2.3. Exclusion Criteria

Clinical cases were excluded if they met any of the following exclusion criteria: (a) the clinical records (including general physical and neurological examination) were incomplete; (b) the results of the bloodwork, MRI studies, or CSF results were incomplete or unavailable for review; or (c) the 7-day outcome and follow-up were unavailable, or the dog received glucocorticoid treatment for more than 24 h before the MUO diagnosis.

### 2.4. Groups

The patients were divided into 2 groups based on their outcome at 7 days post-diagnosis: survived and non-survived. Non-survived dogs comprised dogs who died because of MUO and dogs who were euthanized following a severe clinical deterioration because of MUO.

### 2.5. Data Collection

The data gathered for each dog included their breed, age, sex, weight, institution, duration of steroid therapy before referral to the neurology service, leukocyte and neutrophil counts, concentrations of serum inflammatory parameters (C-reactive protein (CRP), haptoglobin, ferritin, albumin, fibrinogen IgG, IgM, and IgA), paraoxonase-1 (PON-1) activity at the time of presentation, survival status at 7-day post diagnosis, and the reason for death.

### 2.6. Statistical Analysis

The qualitative and dichotomized quantitative variables were summarized using percentages, while the quantitative variables were summarized using the median and range. The differences in the quantitative variables regarding the survived and non-survived dogs were assessed using a Wilcoxon rank-sum test. A non-parametric analysis was performed because of the low number of expected non-survived dogs. The differences between the survived and non-survived dogs for dichotomized quantitative variables were assessed using Fisher’s exact test. The statistical significance was set at *p* < 0.05. The data were analyzed using the statistical software R (https://www.r-project.org/ accessed 27 May 2022).

## 3. Results

### 3.1. Study Population

Fifty-two dogs were diagnosed with MUO within the study period, but four dogs were excluded because they received glucocorticoids for more than 24 h before the diagnosis; therefore, 48 dogs met the inclusion criteria. The breeds consisted of 10 crossbreeds, 7 French bulldogs, 3 Yorkshire terriers, 3 Chihuahuas, 3 Zwergpinschers, 3 Maltese, 3 Pugs, 2 Shih Tzus, 2 Pinschers, 2 Jack Russel terriers, 2 American Staffordshire terriers, and 1 of each of the following: Dogo Argentino, Dachshund, English Cocker spaniel, Labrador retriever, pit bull terrier, toy poodle, toy Schnauzer, West Highland white terrier.

Overall, there were 19 males (40%), two of whom were castrated, and 29 females (60%), 14 of whom were neutered. The median body weight was 7.3 kg (range 1.4–52 kg) and the median age at presentation was 66 months (range 6–144 months).

At the 7-day follow-up period, nine dogs had died (19%). Of those, three dogs died spontaneously because of severe clinical deterioration that was MUO-related, two of whom had progressive seizures and one who showed severe obtundation. The other six dogs were euthanized upon the owner’s request because of failure of clinical improvement or a clinical deterioration despite treatment. Those dogs showed progressive seizures (*n* = 2), obtundation, cranial nerve deficits and multifocal spinal pain (*n* = 1), non-ambulatory tetraparesis (*n* = 1), multifocal spinal pain and tetraparesis (*n* = 1), and severe obtundation and vestibular signs (*n* = 1). The median time to death was 3 days (range 1–7 days). A 3-month follow-up was scheduled for the remaining dogs included in the study and a total of 12 dogs died during this period (25%). Of those, two dogs died spontaneously due to progressive seizures and one was euthanized upon the owner’s request due to failure to improve. Two of the 48 dogs received one dose of glucocorticoids the day before the diagnosis and only one of them died during the 7-day follow-up period. Glucocorticoids were started at a median of 0 days from the MUO diagnosis (range 0–5 days). Only two dogs received the first dose 48 h after the diagnosis, and one started the therapy 5 days post-diagnosis; all survived the 7-day follow-up period. All of the other dogs (*n* = 45) received the first glucocorticoid dose during the first 24 h from the diagnosis.

### 3.2. APPs Analyzed at the Time of Diagnosis in Survived and Non-Survived Dogs

**Figure 1:**
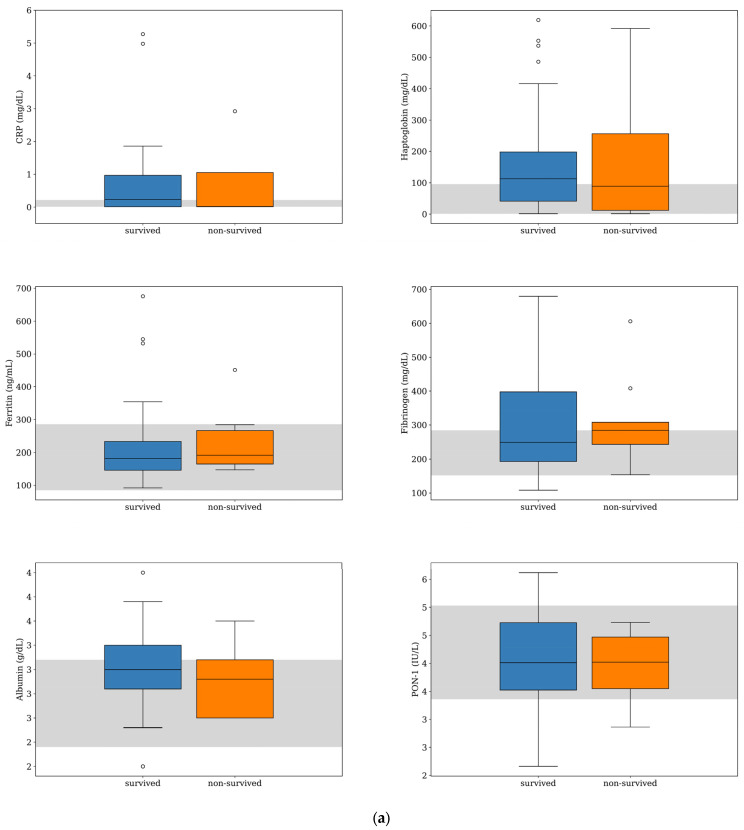

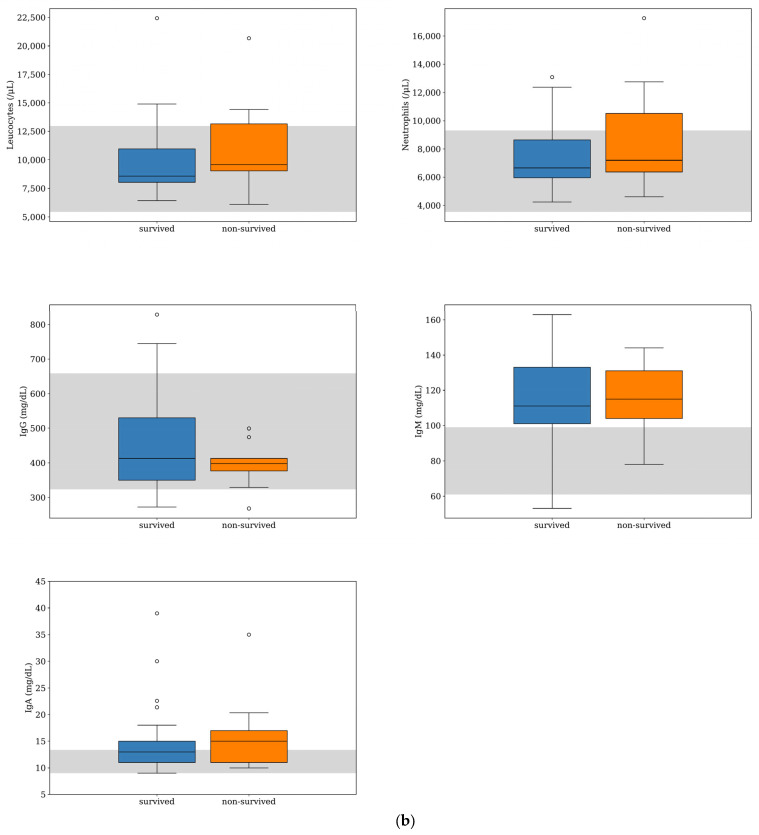
Box-and-whisker plots of acute phase protein concentrations (**a**) and other inflammatory parameters (**b**) evaluated at the time of diagnosis for dogs with MUO that survived (*n* = 39) or did not survive (*n* = 9) to the 7-day follow-up period. For each box, the central horizontal line represents the median, and the lower and upper boundaries represent the 25th and 75th percentiles, respectively. Whiskers represent the most extreme observations that were not outliers. Circles represent outliers (i.e., values that were less than or greater than the 25th or 75th percentile values by >1.5 times the interquartile range). The shaded region represents the reference interval for each parameter. One of the dogs that survived was an extreme outlier (IgA level of 166 mg/dL), but this data point is not shown on the plot for visual clarity.

**Figure 2:**
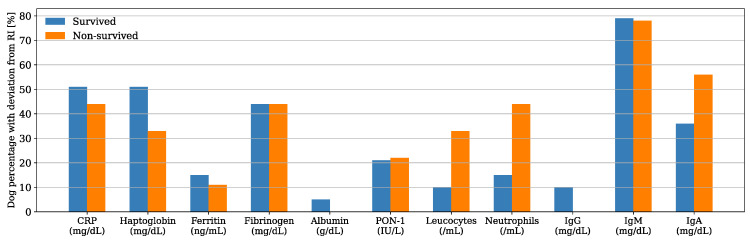
Bar plot showing the percentage of dogs with respect to the group (i.e., x data are equal to n/39·100 or x = n/9·100 in survived and non-survived dogs, respectively) with acute phase proteins (APPs) and other inflammatory biomarkers outside the reference interval (RI).

Serum CRP and haptoglobin were the APPs with the highest deviation from the reference interval (RI) in both survived and non-survived dogs (Figure 1), and their median concentrations were within the RI for the non-survived dogs and slightly above the maximum value of the RI for the survived dogs (Table 1). Nevertheless, their median concentrations were not significantly different between the two groups (CRP, *p* = 0.904; haptoglobin, *p* = 0.704). Similarly, there was no significant difference in the median concentrations of ferritin, fibrinogen, and albumin and the median PON-1 activities between the survived and non-survived dogs. The acute phase proteins most frequently found above the RI were CRP (50%, *n* = 24), haptoglobin (48%, *n* = 23), and fibrinogen (44%, *n* = 21) (Figure 2). The number of dogs with these parameters and other APPs outside the RI was similar between the survived and non-survived groups (Table 2).

### 3.3. Other Inflammatory Biomarkers Analyzed at the Time of Diagnosis in Survived and Non-Survived Dogs

IgA was the other inflammatory biomarker with the greatest deviation from the RI in both survived and non-survived dogs (Figure 1); however, the IgA median concentration was within the RI for the survived dogs and slightly above it for the non-survived dogs (Table 1). There was no difference between the median counts of leukocytes and neutrophils and the median concentrations of IgG, IgA, and IgM between the two groups. The parameters more frequently found above the RI were IgM (79%, *n* = 38) and IgA (40%, *n* = 29), (Figure 2). There was a tendency for the neutrophils count to be more frequently found within the RI in the survived dogs; however, the difference was not statistically significant (*p* = 0.075) (Table 2). The number of dogs with a leukocyte count, IgG, IgA, and IgM outside the RI was similar between the two groups (Table 2).

## 4. Discussion

The aim of this study was to describe the behavior of serum and plasma inflammatory parameters in dogs newly diagnosed with MUO and to evaluate their usefulness as potential predictors for 7 days of survival. This 1-week period of time was chosen based on the previously reported higher mortality in dogs with MUO in the first few days following diagnosis, regardless of the type of immunosuppressive protocol initiated [4,13,19]. In the previous literature, the mortality rate ranges from 10% to 33% during the first 7 days following diagnosis and from 10% to 56% during a period of 3 months post-diagnosis [4,13,14,17,19,27]. The 19% and 25% mortality rate at 7 days and 3 months, respectively, of the present study corroborates the previous research.

This study suggests that there is no correlation between the inflammatory parameters evaluated and the outcome of MUO during a period of 7 days post-diagnosis between survived and non-survived dogs. The inflammatory cells evaluated were the leukocytes and the neutrophils. A previous study evaluating 1-week survival in dogs with MUO found a correlation between a higher CSF neutrophil count and mortality but failed to find a relationship between blood leukocyte or neutrophil counts and the short-term outcome [20]. The latter result was also confirmed by our study. Moreover, only 21% (10/48) of the patients in the present study had a serum neutrophil count above the RI at presentation, and the neutrophil count was unable to predict the short-term outcome. On the other hand, steroid-responsive meningo-arteritis (SRMA), which is the second most common presumed immune-mediated inflammatory CNS disease after MUO [1], is typically characterized by an inflammatory leukogram with left-shift neutrophilia, along with neurological symptoms. [28]. In human medicine, the cause of increased neutrophil counts in CNS autoimmune disease and their role in the pathophysiology of the development of CNS autoimmunity has yet to be clarified [29]. In humans, autoimmune encephalitis (AE) is a heterogeneous group of inflammatory CNS disorders considered similar to MUO [30]. Previous studies on a mice model of experimental autoimmune encephalomyelitis (EAE) demonstrated an important role for neutrophils in promoting brain inflammation by showing that the Ab-mediated depletion of neutrophils before the disease induction in mice prevented the development of inflammation of the brain [30]. In addition, it seems clear that neutrophils play a role in promoting CNS parenchymal infiltration of leukocytes, especially in the brain compared to the spinal cord, and they may be particularly important early in the disease and in lesion formation during relapses [31]. In AE, an increased blood neutrophil count is usually present; furthermore, the neutrophil-to-lymphocyte ratio and monocyte-to-lymphocyte ratio are significantly correlated with the severity of the disease [32]. In our study, most dogs did not display an increase in serum neutrophil count, and the neutrophil-to-lymphocyte ratio and the monocyte-to-lymphocyte ratio were not evaluated.

APPs have been previously investigated as biomarkers of systemic inflammation in dogs with SRMA [28]. A significant increase in serum and CSF CRP concentrations and a decrease in the serum albumin concentration have been shown in dogs with SRMA [28]; however, APPs behavior in MUO has been investigated only in one previous study comparing CSF and serum D-dimer and CRP concentration between groups with various neurological diseases [33]. In this previous study, the inflammatory neurological disease group comprised mostly (29/37) MUO cases, but no variation in the serum and CSF concentrations of CRP or D-dimer were found between the MUO cases and most of the other groups, except for the SRMA group, in which the CSF CRP and D-dimer concentrations were significantly higher [33]; however, in the present study, 50% (24/48) of the dogs included had CRP values above the RI at presentation.

Other APPs found to be elevated are haptoglobin and fibrinogen. Nevertheless, the 44 to 50% of dogs with an increase in these APPs value in the present study is not considered worthy of value and, as we do not have a control group of sick dogs with a similar presentation, any conclusion on the usefulness of CRP and other APPs as biomarkers of MUO is difficult to be drawn; therefore, the importance of APPs as biomarkers of MUO has yet to be proven [21] and, based on our results, they do not seem to play a role in predicting the short-term outcome of MUO.

The concentration of distinct serum immunoglobulin families was evaluated in the present study: IgG, IgA, and IgM. Serum and CSF immunoglobulins were previously investigated in a small group of dogs with MUO (5/69) relative to other infectious and immune-mediated inflammatory CNS diseases [34], showing that immunoglobulins were generally elevated in dogs with MUO. A similar result can be found in our study, where IgM was above the RI in 79% (38/48) of the patients, followed by IgA in 40% (19/48), while the IgG was mostly in the RI. It should be noted that in the previous study, no significative differences were found amongst the groups with different inflammatory CNS diseases; therefore, the immunoglobulins were not considered a good marker of MUO, as they lacked specificity. Conversely, in dogs with SRMA, IgA has proved to be a valuable biomarker, especially when evaluated in serum and CSF [28]. Moreover, in humans, it has been shown that distinct families of immunoglobulins are involved in different diseases; however, contrary to what our study may suggest, specific Igs more frequently elevated in AE are the IgG family, and the detection of specific IgA and IgM in AE is considered to be of unclear significance [35]. Finally, all the specific Igs considered in our study failed to predict the short-term outcome.

From the results of the present study, we can state that contrary to SRMA [28], in dogs with MUO, no consistent systemic inflammatory condition was detectable in any of the patients; however, there is a tendency for some inflammatory parameters to be elevated in a variable proportion of dogs with MUO. These results are in agreement with the findings that MUO symptoms are usually confined to the CNS, while dogs with SRMA frequently show pyrexia [36,37], and other non-neurological signs can also be present, such as arrhythmias secondary to myocarditis [38], and reluctance to walk, lameness and appendicular joint pain due to concomitant polyarthritis [39], suggesting a more systemic inflammatory condition.

Because of its retrospective nature, this study has some limitations. Although the treatment protocol is relatively standardized in our practice, the initial decisions made by individual clinicians were also based on specific patient necessities. Since the outcome could be influenced by the therapy chosen, some serum parameters could have failed to predict the outcome because of the influence of the immunosuppressant used. Furthermore, glucocorticoid therapy was started only 2 and 5 days after the diagnosis in two and one dogs. This delay in starting immunosuppressive treatment was caused by the need to wait for infectious disease testing results, especially in cases where the diagnosis of MUO was uncertain. This applies, for example, to dogs who did not undergo a CSF tap or whose results came back normal. Including these dogs in the study could be a limitation, as the delay in starting therapy could have impacted the outcome; however, these dogs managed to survive the 7-day follow-up period, which was used to assess variations in the blood parameters. As a result, the study’s findings are most likely accurate. Moreover, as 6/9 (67%) of dogs in the non-survived group died because they were euthanized, there is a chance that some of these patients would have survived more than the 1-week time frame. These patients were euthanized only because of severe neurological signs; therefore, the prognosis was reserved/guarded. Furthermore, it should be noted that a conclusive diagnosis could not be reached for the patients who did not survive. This is a limitation, as the most accurate diagnosis for MUO still requires histopathology [2,3,11]. Unfortunately, consent for post-mortem analysis was not obtained for any of the dogs that passed away during this study; however, our inclusion criteria make other differential diagnoses very unlikely.

## 5. Conclusions

In conclusion, this study is the first to focus on routinely assessed serum inflammatory markers in dogs diagnosed with MUO. Higher concentrations of IgM, IgA, CRP, haptoglobin, and ferritin are not consistently present in dogs with MUO, and these biomarkers are not useful for predicting the short-term outcome of this disease; thus, we suggest that future research should look for other variables to guide the clinician in predicting the outcome of this complex disease.

## Figures and Tables

**Table 1:** Reference interval (RI) of the inflammatory variables analyzed and median counts, median concentrations, and median activity of the same inflammatory variables between survived and non-survived dogs.

Variable	RI	Survived (*n* = 39)	Non-Survived (*n* = 9)	*p*-Value
CRP (mg/dL)	0.01–0.22	0.24 (0.01–5.27)	0.02 (0.01–12.76)	0.904
Haptoglobin (mg/dL)	1–96	113 (1–619)	89 (1–592)	0.704
Ferritin (ng/mL)	85–286	182 (92–676)	192 (147–451)	0.465
Fibrinogen (mg/dL)	152–284	249 (108–680)	284 (154–606)	0.749
Albumin (g/dL)	2.7–3.6	3.5 (2.5–4.5)	3.4 (3-0–4.0)	0.603
PON-1 (IU/L)	3.86–5.53	4.51 (2.66–6.12)	4.52 (3.36–5.23)	0.728
Leukocytes (/μL)	5450–12,980	8570 (6420–22,430)	9590 (6090–20,670)	0.342
Neutrophils (/μL)	3555–9314	6660 (4240–1390)	7200 (4610–17,260)	0.271
IgG (mg/dL)	323–659	413 (272–829)	398 (268–499)	0.535
IgM (mg/dL)	61–99	111 (53–163)	115 (78–144)	0.826
IgA (mg/dL)	9–13.4	13 (9–166)	15 (10–35)	0.503

Data are reported as median and range. CRP, protein reactive-C; PON-1, paraxonase-1.

**Table 2:** Total number and number of dogs with the inflammatory variables outside the reference interval between survived and non-survived dogs.

Variable	Total (*n* = 48)	Survived (*n* = 39)	Non-Survived (*n* = 9)	*p*-Value
Dogs with CRP concentration above RI	24 (50%)	20 (51%)	4 (44%)	0.151
Dogs with haptoglobin concentration above RI	23 (48%)	20 (51%)	3 (33%)	0.466
Dogs with ferritin concentration above RI	7 (15%)	6 (15%)	1 (11%)	1
Dogs with fibrinogen concentration above RI	21 (44%)	17 (44%)	4 (44%)	1
Dogs with albumin concentration below RI	2 (4%)	2 (5%)	0 (0%)	1
Dogs with PON-1 activity below RI	10 (21%)	8 (21%)	2 (22%)	1
Dogs with leukocytes count above RI	7 (15%)	4 (10%)	3 (33%)	0.111
Dogs with neutrophils count above RI	10 (21%)	6 (15%)	4 (44%)	0.075
Dogs with IgG concentration above RI	4 (8%)	4 (10%)	0 (0%)	1
Dogs with IgM concentration above RI	38 (79%)	31 (79%)	7 (78%)	1
Dogs with IgA concentration above RI	29 (40%)	14 (36%)	5 (56%)	.451

CRP, protein reactive-C; PON-1, paraxonase-1; RI, reference interval. Data are reported as numbers and percentages.

## Data Availability

The data presented in this study are available on request from the corresponding author upon reasonable request.
